# Insights into the Mode and Mechanism of Interactions Between RNA and RNA-Binding Proteins

**DOI:** 10.3390/ijms252111337

**Published:** 2024-10-22

**Authors:** Yan Fang, Xinyu Liu, Yuli Liu, Naiyi Xu

**Affiliations:** College of Animal Science and Technology, Southwest University, Chongqing 400715, China; yanfang003@outlook.com (Y.F.); liuxinyu06162006@126.com (X.L.); liuyuli2819@126.com (Y.L.)

**Keywords:** RNA-binding protein, gene expression and regulation, human disease

## Abstract

Both RNA and protein play important roles in the process of gene expression and regulation, and it has been widely discussed that the interactions between RNA and protein affect gene transcription, translation efficiency, and post-translational modification. As an important class of proteins, RNA-binding proteins bind to RNA and affect gene expression in various ways. Here, we review the structural and functional properties of RNA-binding proteins and illustrate the specific modes of interactions between RNA and RNA-binding proteins and describe the involvement of some representative RNA-binding protein families in this network of action. Furthermore, we also explore the association that exists between RNA-binding proteins and the onset of diseases, as well as their potential in terms of serving as a therapeutic tool for the treatment of diseases. The in-depth exploration of the interactions between RNA and RNA-binding proteins reveals the dynamic process of gene expression and regulation, as well as offering valuable insights to advance the progress in the dissection of disease mechanisms and research and discovery of drugs, which promote the development of molecular biology.

## 1. Introduction

RNA plays an important role in gene expression and regulation, not only through mRNAs involved in the process of transcription and translation, but also through non-coding RNAs (ncRNAs) such as microRNAs (miRNAs) [1,2,3,4,5], small interfering RNAs (siRNAs) [6,7], long non-coding RNAs (lncRNAs) [8,9], etc. These RNA molecules impact gene expression and function through different pathways and mechanisms, participating in various biological processes within the organism. Therefore, the research into RNA-regulated gene expression processes contributes to the deeper understanding of the complexity and diversity of gene expression regulation, as well as providing novel ideas for the genetic engineering and even treatment of diseases. Meanwhile, proteins play a variety of key roles in gene expression and regulation, ranging from participating in the fundamental processes of gene expression, such as transcription and translation, to acting as transcription factors [10,11], epigenetic regulators [12,13], and signaling molecules. Altogether, these processes involving proteins contribute significantly to the accurate transmission of genetic information and the refined regulation of expression.

Between RNA and protein exists a tight and complex connection, which is reflected in the transmission of genetic information and in the interaction between them. Nearly all RNA depends on interactions with proteins to perform their functions completely, while proteins require RNA binding to fulfill regulatory roles [14]. The interaction between RNA and protein in gene expression and regulation is multi-pathway, such as they collaborate to regulate gene expression and cellular functions through direct binding and participation in post-transcriptional modifications. Among the complex class of proteins that interact with RNA, RBPs are key effectors of gene expression, which recognize hundreds of transcripts and form extensive regulatory networks that contribute to the maintenance of homeostasis in the intracellular environment [15]. The dynamic interactions between RNA and RBPs within ribonucleoprotein are a cornerstone of RNA biology [16]. Numerous cellular processes including gene expression and regulation and RNA processing and decay, as well as protein localization, would be inconceivable without the direct and specific interactions between RNA and RBPs [17]. To be more specific, RNA-RBP interactions are primarily manifested in the regulation of RBPs for all aspects of RNA life, including splicing, export, translation, and stability. Furthermore, RNAs may modulate the activity of RBPs [18] or determine their binding affinity to RBPs [19]. In fact, to deepen the understanding of the linkage between RNA and RBPs, it is essential to elucidate the RNA-RBP interactions during gene expression. The binding of RBPs to RNA is the key to regulating the gene expression network, with RNA-binding domains (RBDs) serving as the functional units responsible for binding RNA. Currently, the research on RBPs mainly focuses on their potential in the development of various diseases and lack of RNA-RBP interactions. In this review, we discussed the binding process of RNA and RBPs from a structural point of view, and systematically elaborated on the specific process of how the binding affects gene expression and regulation. In order to present the significance of RNA-RBP interactions in normal life activities, we also illustrated the connection between human diseases and RNA-RBP interactions to provide ideas and a theoretical basis for disease treatment.

## 2. Structural and Functional Characteristics of RBPs

RBPs are a group of proteins that widely exist in organisms, whose main function is to bind to RNA molecules and participate in a variety of lifecycle processes of RNA. Due to the unity of structure and function, RBPs usually contain some specific structural domains or motifs that are essential for their binding to RNA. RBPs were initially identified because of their ability to bind various types of RNAs by forming stable secondary and tertiary structures of RBDs [20]. Upon binding to RNA, these RBPs alter the fate of the bound RNA and extensively control every stage of the RNA lifecycle, including mRNA synthesis and maturation, activation, splicing, and degradation of ncRNA. The majority of the study on classical sequence-specific RBDs focuses on the hnRNP-K-Homology (KH) domain, RNA recognition motifs (RRMs), and zinc finger (ZnF) domains [21,22,23]. In addition, there are many unconventional RBPs (ucRBPs), proteins that can bind to RNA but lack known RBDs. The vast majority of ucRBPs have uncharacterized RNA-binding specificity, while a small percentage of ucRBPs also display well-defined sequence specificity [24]. Interestingly, a number of RNA-binding sites were identified in ucRBPs, the enzymes peptidyl-prolyl cis-trans isomerase, enolase 1, and phosphoglycerate kinase [25]. These ucRBPs exert their molecular functions in different capacities, including cell cycle regulators, metabolic enzymes, protein scaffolds, and antiviral factors [26], which are responsible for cell cycle regulation, RNA metabolism, and signaling. And their specific structural characteristics are not yet well understood due to the most uncharacterized motifs. RBPs can participate in the processes of RNA splicing, mRNA export, translation, and stability through their recognition and targeted binding of specific motifs to RBDs. In addition, non-coding RNAs in turn affect the function of RBPs by binding to RBDs. The specific functions of RBPs are mediated through the relevant binding activities between RNA and RBDs, and the interactions between RNA and RBPs are likewise contingent upon these RBDs. The study of an RBP structure helps us to gain a deeper understanding of the role of RBPs in RNA biology, and the following section will focus on the three classical RBDs of RBPs and ucRBPs.

### 2.1. KH Domain

**Figure 1:**
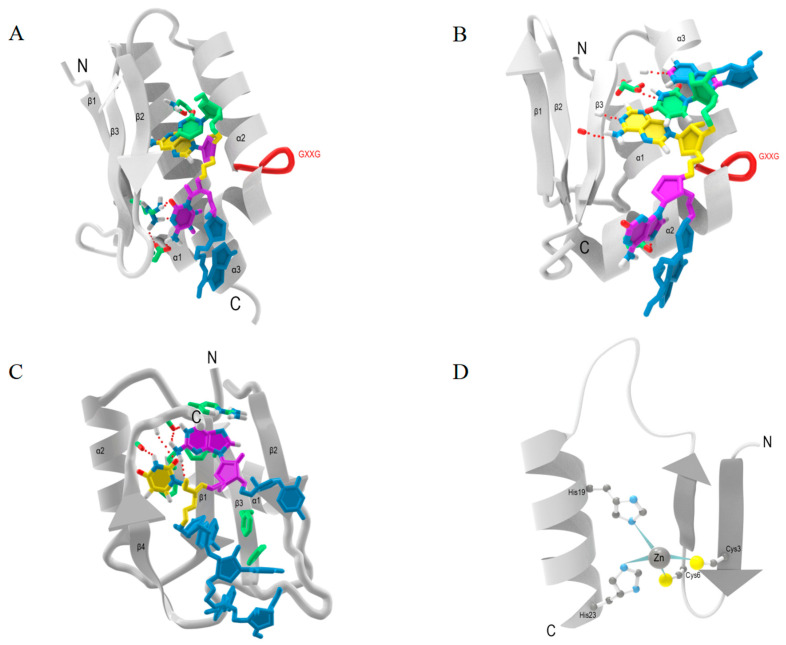
The structure of three classical RBDs including hnRNP-K-Homology (KH) domain, RNA recognition motif (RRM), and zinc finger (ZnF) domain. (**A**) Type I KH domain, the additional α and β elements are located at the C-terminus. (**B**) Type II KH domain, the additional α and β elements are located at the N-terminus. (**C**) RNA recognition motif (RRM) has a βαββαβ topology, forming a four-stranded β-sheet stacked on two α-helices. (**D**) ZnF domain of CCHH type. Zinc is bound by four coordination bonds with two Cys and two His residues, forming a finger-like structure. The proteins are shown as the gray ribbons; RNA-contacting side chains and nucleotides are represented by other colors.

The KH domain was first found in heterogeneous nuclear ribonucleoprotein K (hnRNP K) [27]. The KH domain, a 70 amino acids domain, can bind to single-stranded DNA and single-stranded RNA, and is ubiquitous in eukaryotic, bacterial, and archaea. There is an important characteristic sequence in the center of the domain-(I/L/V)-I-G-X-X-G-X-(I/L/V) [28,29]. Due to the lack of aromatic amino acid, the recognition between KH and RNA is achieved by the function of hydrogen bonding, electrostatic interactions, and shape complementation [20]. Structurally, the KH domain is elegantly arranged, consisting of three-stranded β sheets stacked on three α helices, and a minimal βααβ core is commonly presented. According to the different positions of the additional α and β elements, the HK domain is divided into two types; in the type I KH domain, the α and β elements are located at the C-terminus (Figure 1A), whereas in the type II KH domain, these elements are located at the N-terminus (Figure 1B) [23]. KH domains present in RBPs help in RNA recognition and RNA binding [30]. The highly conserved dual KH domains in MEX-3 proteins enable RNA binding and are essential for the recognition of the 3′-UTR and post-transcriptional regulation of MEX-3 target transcripts [31]. This suggests that the process of RNA recognition by the KH domains underlies the regulation of gene expression by RBPs. Poly (C)-binding proteins (PCBPs) containing three type I KH domains bind to poly (C)-rich RNA sequences and play a role in mRNA stabilization, translational activation, and translational silencing [32]. The mechanisms through which certain proteins execute their functions are largely contingent upon the involvement of KH domains. Bicc1 is a conserved RBP that represses the translation of selected mRNAs. It was found that the KH domains are essential for its RNA binding, especially the second of its KH domains, KH2, which is the only domain necessary for the function of repression [33]. The RBP KSRP contains four KH domains, which serve to promote specific mRNA degradation. The mRNA-binding and mRNA degradation activities of KSRP are closely related to the conserved structural elements of its fourth KH domain (KH4), while the KH4 requires the third KH domain (KH3) for its function in mRNA recognition and decay [34]. In addition, problems with the binding of KH domains to RNAs can cause disease. For example, mutations in the KH domain of FMR1 lead to impaired RNA binding, resulting in fragile X syndrome [30]. It has also been demonstrated that some RBPs containing the KH domain work with miRNA to regulate gene expression [35], which indicates that the KH domain is likely to play an important role in gene expression regulation in coordination with miRNA pathways.

### 2.2. RRM

An RNA recognition motif (RRM), consisting of 80–90 amino acids, is the most common RNA binding module [22]. Characterized by a βαββαβ topology, the RRM folds into a four-stranded β-sheet stacked on two α-helices (Figure 1C). Among them, the β sheet is the main RNA binding surface, typically interacting with single-stranded RNA [36]. Three conserved residues, an Arg or Lys residue as well as two aromatic residues located on the β-sheet, allow recognition of nucleotides on the RNA by salt-bonding and aromatic stacking interactions, which are located in two highly conserved motifs [22]. Additionally, a single RRM typically recognizes 4–8 nucleotides using exposed loop structural elements [20]. During pre-rRNA and pre-mRNA processing events such as splicing, 3′-end processing, stability, and transporting [37], it is inseparable from the function of an RRM. For example, hnRNP L contains four RRMs, which contribute to RNA binding to CA repeats or CA-rich elements. The β-sheets of RRM1 and RRM34 can be used for RNA binding. Moreover, RRM34 promotes RNA looping upon binding to two appropriately segregated binding sites within the same target pre-mRNA. The above processes fulfill the function of hnRNP L as an activator or repressor in the regulation of alternative splicing [38]. Therefore, RRMs play a major role in the post-transcriptional regulation of gene expression. Human RRM-containing RBPs are classified as RNA metabolism proteins, with a considerable enrichment in two functional pathways including spliceosome and mRNA surveillance. Further, 18% of the RRM-containing RBPs are implicated in various human diseases [39]. It is speculated that an RRM is likely to serve as a binding site for the treatment of human diseases. An RRM is an essential structural component for the function of RRM-containing RBPs. Whereas RRMs at different positions of the same protein play diverse functions in the process of recognition and binding to RNA, most of them exist in synergy with each other [40,41], acting together in the binding process and ultimately affecting transcription and translation.

### 2.3. ZnF Domain

The ZnF domain, as a classical DNA-binding protein domain [42,43], can also bind to RNA. Usually, it is categorized according to the type of residues used to coordinate zinc such as CCHH/CCCH/CCHC and so on [44], of which CCHH is the most common type (Figure 1D) [45]. The ZnF domain is rarely found alone; it tends to form tandem arrays [46] and exist as multiple repeats in the protein. Transcription factor TFIIIA, the first zinc finger-containing protein, was initially discovered through its association with 5S rRNA, which contains nine ZnFs of CCHH type [47,48]. The classic CCHH ZnF usually contains 28–30 repeating amino acid sequences, including two conserved cysteine and two conserved histidine residues [49]. CCHH ZnF interacts with RNA through the formation of direct hydrogen bonds with Watson–Crick base pairs in major grooves [20]. After the coordination of Zn, the zinc finger folds into a structure consisting of an α-helix and an antiparallel β-sheet, which can function as a transcription factor by recognizing and binding to specific DNA targets [50]. In addition, residues in the α-helix played a key role in the recognition process, because the identification of a specific DNA sequence is achieved by the interaction of DNA bases and side chains on the α-helix’s surface [51]. TFIIIA binds to RNA through the helix of the ZnF combining with the RNA loop specially [52,53]. Thus, the ZnF domain can recognize RNA by a similar principle and method to that described above, but the different RNA structures determine the unique structural arrangement of the ZnF on the nucleic acid template. The current exploration of the ZnF in RBPs has been limited to the structural level, with less attention paid to its role in gene expression and regulation. Nuclear Poly(A) RNA-binding Protein 2 (Nab2), consisting of three tandem Cys-Cys-Cys-His (CCCH) zinc fingers, is a polyadenosine RNA-recognizing domain, which is essential for proper mRNA 3′-end formation [54]. It is demonstrated that the ZnF domain plays a fundamental guarantee in the complex processes of performing the functions of the related RBPs.

### 2.4. ucRBPs

Interactome capture in the past decade has identified ucRBPs that lack classical RBDs to interact with RNAs including metabolic enzymes, transcription factors, heat shock proteins, and kinases as well as several chromatin-associated proteins [55]. Their mode of binding to RNA includes binding by intrinsically disordered protein regions (IDRs), shape complementarity, and metabolic enzymes [25,56]. Among them, there are more studies on binding by metabolic enzymes, with some enzyme–RNA interactions having a role in gene regulatory feedback. For example, Thymidine synthase establishes a negative feedback loop by binding to its own mRNA, thereby inhibiting translation in the absence of its substrate [57]. Aconitase, as a cytoplasmic iron-regulatory protein 1 (IRP1), binds to iron-deficient cells, and interaction with an iron-responsive element (IRE) blocks translation of Ferritin mRNA, whereas binding to IRE in the 3′ UTR leads to mRNA stabilization [58]. In addition, these metabolically functional RBPs can be associated with disease if disturbed. For instance, the mutation in the binding between inosine 5′-monophosphate dehydrogenase (MPDH) and RNA can influence the translation of rhodopsin mRNA, which is accompanied by severe visual impairment [57]. Furthermore, the binding activity of some ucRBPs has been shown to be essential for virus infection, which demonstrates the potential of ucRBPs in host-based therapies against viruses [59]. It is not difficult to see that ucRBPs have their own patterns in the regulation of gene expression, and in-depth study of ucRBPs at binding sites vital to mRNA regulation is very necessary, which is of great significance to the understanding of related disease mechanisms and the development of therapies.

## 3. Interactions Between RNA and RBPs

**Figure 2:**
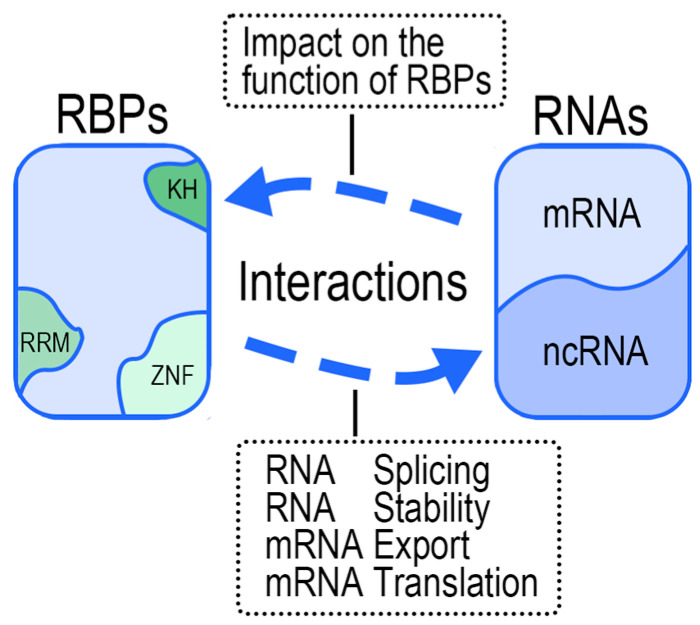
The interactions between RNAs and RBPs. The realization of the interactions between RNA and RBPs is largely dependent on the binding between them. RBPs can perform their specific biological functions, including RNA splicing, RNA stability, mRNA export, mRNA translation, etc., by binding to specific RNA-binding domains, such as hnRNP-K-Homology (KH) domain, RNA recognition motif (RRM), and zinc finger (ZnF) domain. RNAs can also act as decoys, scaffolds, or guides to regulate the functions of RBPs by influencing the modification and localization of their binding proteins.

The interaction between RNA and RBPs is orchestrated by a suite of molecular forces that facilitate the binding of RBPs to their target RNA. This binding is mediated through chemical interactions between RBDs and RNA nucleotides, encompassing hydrogen bonding, Van der Waals interaction, and hydrophobic and π interactions and stacking [60], which ensure stable binding between the two. This process is also the basis for further roles in gene expression and regulation. RBDs are the key site for the interaction between RNA and RBPs, and influence gene expression and regulation, which are also dependent on specific molecular pathways in transcriptional and translational events. As the primary post-transcriptional controllers of RNA export, RBPs coordinate a myriad of fundamental processes in protein-coding gene expression, such as co-transcriptional pre-mRNA processing events [61], post-transcriptional degradation of RNA, and its downstream gene expression (Figure 2) [62]. Testis–brain RNA-binding protein (TB-RBP), a testis translationally regulated RBP, binds to the 3′ untranslated region of transporter brain mRNAs and plays a role in mRNA storage, translocation, and localization in the brain and testis [63]. Two RBPs, SRSF1 and HNRNPU, have been identified that regulate lipopolysaccharide-induced alternative pre-mRNA splicing of the MyD88 signaling transporter [64]. In addition, there is a strong association between the binding location of the RBP and its function (Figure 2). The binding to translation termination sites and 3′ untranslated regions is important to most human RBPs in the regulation of RNA decay [65]. Therefore, RNA splicing, transcription and translation efficiency, stability, and other processes are likely to be variously affected by RBP binding, and the network of relationships formed by RNA and RBPs also plays an important role in the gene expression and regulation. The mode of interaction under the RNA-RBP network will be introduced specifically below.

### 3.1. Role of RBPs in RNA Alternative Splicing

**Figure 3:**
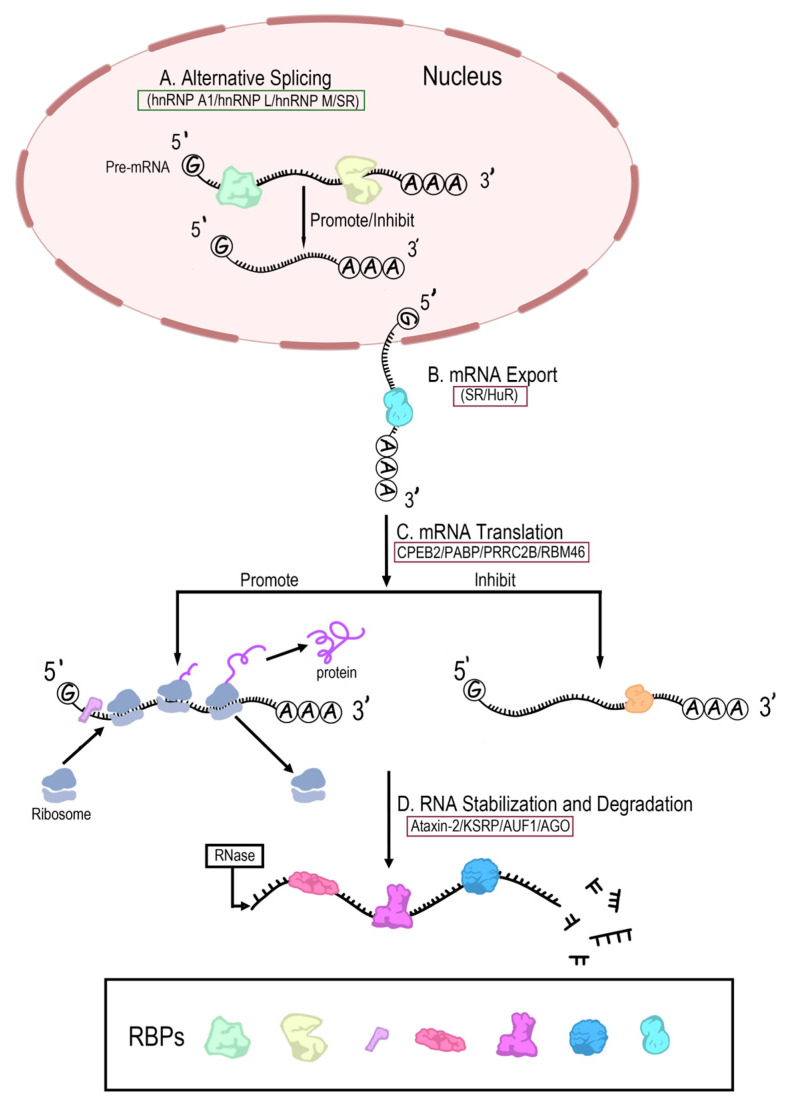
mRNA-RBP interaction in gene expression and regulation. It consists of four main processes: RNA alternative splicing, mRNA export, mRNA translation, and RNA stabilization and degradation. Alternative splicing is a fundamental step in mRNA maturation that regulates gene expression and maintains proteome diversity, and is regulated by multiple RBPs (e.g., hnRNP A1, hnRNP L, hnRNP M, SR). By shuttling between the nucleus and cytoplasm, some RBPs (e.g., SR, HuR) form ribonucleoprotein complexes that help mature mRNAs to be exported from the nucleus to the cytoplasm. RBPs fulfill the purpose of promoting or inhibiting the translation of RNA by binding to the appropriate sites of RNA. In addition, RBPs can form stable complexes with RNA to protect it from degradative enzymes, and they can also form complexes with RNA with specific degradative activities to promote degradation. mRNA-RBP interactions usually affect gene expression and regulation through the four processes described above.

RNA alternative splicing refers to the process of generating different mRNA splicing isoforms from the pre-mRNA by different splicing methods that choose different splicing sites. Pre-mRNA splicing, a fundamental step of mRNA maturation [66], is an important mechanism for the regulation of gene expression and diversification of the proteome [67], which can generate multiple proteins from a single gene. Therefore, the number of proteins in the proteome exceeds the number of genes in the genome. Notably, 95% of human genes undergo alternative splicing [68], underscoring its biological importance. The regulation of alternative splicing is mediated by specific RBP motifs and their corresponding RNA binding sites (Figure 3). A critical family of RBPs, hnRNP, binds to the pre-mRNA in the nucleus and exerts a substantial influence on RNA processing and splice site selection [69]. HnRNP has been identified as containing four unique RBDs: the RRM, the quasi-RRM, a glycine-rich domain containing an RGG box, and a KH domain [70]. Specifically, hnRNP A1 counteracts the splicing activity of splicing factors such as SF2/ASF or SC35, leading to activation of the distal 5′ splice sites. The two conserved phenylalanine residues in its two RRMs are likely involved in specific RNA–protein interactions and are essential for alternative splicing regulation [71]. And hnRNP A1 interacts directly with mRNA in its 3′UTR region, augmenting the translational capacity while maintaining mRNA stability [72]. HnRNP L predominantly regulates cassette-type alternative splicing [73] and has been shown to hinder U2AF65 recognition of the 3′ splice site, as well as inhibit splicing by obstructing 5′ splice site recognition of the U1 snRNP [74]. HnRNP M binds to ISE/ISS-3 in a sequence-specific manner, which contributes to its splicing regulatory function [75]. Clearly, alternative splicing can be regulated by multiple RBPs, and this regulatory process is also a crucial step in the maturation of transcribed mRNAs into functionally active mRNAs.

### 3.2. Role of RBPs in mRNA Export

Pre-mRNA splicing is followed by the ushering in of mature mRNA export to the cytoplasm [76]. mRNA export is a critical process in which mRNAs are subjected to alternative polyadenylation to achieve biological activity. During post-transcriptional RNA export, RBPs play important roles in alternative polyadenylation and mRNA transport (Figure 3). ALYREF, an important RBP, plays crucial roles in nuclear export and 3-end processing of polyadenylated mRNA [77], after which a stable mRNP is formed and eventually transported into the cytoplasm. Additionally, the RNA-binding protein human antigen R (HuR) is expressed in virtually all tissues, and mainly located in the nucleus involving mRNA export, which requires forming nuclear HuR-mRNA complexes through binding to target mRNAs of AU-rich elements (AREs) in order to be exported to the cytoplasm [78]. Binding of HuR to target mRNAs can also influence many aspects of mRNA processing, including splicing, polyadenylation, intracellular transport, and so on [79]. Notably, SR proteins, a class of RBPs rich in serine/arginine (S/R) repeats, play roles in RNA splicing [80,81] and mRNA export from the nucleus. They may rely on stable RNA binding mediated by RRMs for shuttling between the nucleus and cytoplasm [82]. In addition, certain SR proteins function as adaptor proteins for mRNA export by interacting with the export receptor TAP/nuclear export factor 1 (NXF1) [83]. Generally speaking, RBPs are involved in the formation of ribonucleoprotein complexes and mRNA recognition and packaging to ensure normal gene expression.

### 3.3. Role of RBPs in mRNA Translation

RBPs can regulate translation initiation, elongation, and termination. RBPs are able to influence mRNA translation by regulating translation initiation factors (Figure 3). mRNA-binding proteins generally present in the cytoplasm, which facilitate ribosome binding through a 5′-end, cap-mediated mechanism to prevent false initiations at aberrant translation initiation sites [84]. Notably, the mRNA translation requires the activation of eukaryotic initiation factors (eIFs) to recognize the mRNA’s m7G cap structure at the 5′ end or the poly(A) tail at the 3′ end [85], which means that mRNA-binding proteins can regulate eIFs to affect the translation process. Although RBPs can activate or inhibit translation in principle, they inhibit translation by binding to the 3′-UTR [86]; specifically, RBPs bind to cis-acting elements in 3′-UTR by targeting the eIF4E-eIF4G complex to regulate mRNA translation [87], and RBPs can regulate translation elongation and termination. Cytoplasmic polyadenylation element-binding protein (CPEB) 2 has been shown to interact with eEF2 to delay the elongation of the CPEB2-bound RNA peptide [88]. Poly (A)-binding protein (PABP) can bind to eukaryotic release factor (eRF) to recruit eRF3a and eRF1 to the ribosome to promote the termination of translation [89]. Additionally, RBPs can interact with each other to regulate the translation. The 3′ UTR-binding proteins generally inhibit translation through the formation of a closed loop with 5′ cap-binding proteins and intermediate bridging proteins [90]. Recently, with the development of high-throughput sequencing technology and bioinformatics, more and more RBPs have been identified and have been demonstrated to play an important role in mRNA translation. Proline-rich coiled-coil 2B (PRRC2B), a novel RBP, is necessary for the efficient translation of specific proteins required for cell cycle and proliferation [91]. The RNA-binding motif protein 46 (RBM46) regulates the translation of cohesin complex subunits (CCSs) during meiosis by binding to the 3′UTR region [92].

### 3.4. Role of RBPs in RNA Stability and Degradation

For the process of gene expression and translation, mRNA and ncRNA are affected by the stability of RNA. Different RBPs can play roles in maintaining RNA stability and promoting RNA degradation (Figure 3). In terms of maintaining RNA stability, some RBPs are able to bind to specific sequences on RNA to form stable RNP complexes, which protect RNA from degradation by degrading enzymes. Ataxin-2 binds directly to the 3′UTR of target mRNAs, recognizing U-rich elements as binding motifs to stabilize a subset of mRNAs [93]. Now, RNA degradation has also received a lot of attention. RBPs bind cis-regulatory elements with specific RNA sequence or structural motifs to regulate transcript degradation by multiple mechanisms, including decapping of the 5′ end, deadenylation of the 3′ end, and so on [94]. Some of the KH domains of the KH-type splicing regulatory protein (KSRP) have the ability to bind ARE, promoting rapid mRNA decay [95]. mRNA decay protein AUF1 achieves its degradation effect by targeting specific mRNA-containing AREs [96]. RBPs can bind to relevant RNA to form complexes, with specific degradation activities. Members of the Argonaute (AGO) family form RNA-induced silencing complexes (RISCs) with small RNAs, which target complementary RNAs through the small RNAs they bind to and inhibit gene expression through mRNA degradation [97].

### 3.5. Interactions Between RBPs and ncRNA

The interaction between RBPs and ncRNA can also regulate gene transcription and translation. The majority of ncRNAs operate as RNA–protein complexes, such as ribosome, snRNP, snoRNP, telomerase, microRNAs, and long ncRNAs [98]. miRNAs bind to their RBP partners to form an RISC that inhibits mRNA translation [99]. m6A readers can be used as RBPs to recognize and target the lncRNAs modified by m6 A, and to regulate the degradation and transcription of lncRNAs [100,101].

It is notable that, contrary to conventional belief, RNAs also regulate a variety of biological processes within the cell through modulating the function of RBPs [25]. lncRNAs act as decoys, scaffolds, or guides for RBPs in epigenetic regulation and influence the modification, stability, localization, and activity of the proteins they bind to, thereby regulating genes at the transcriptional and translational levels [100]. lncRNA CECR7 enhances the stability of EXO1 by the recruitment of RBP U2AF2, which promotes the development of hepatocellular carcinoma [102]. In addition, circRNAs are able to change the RBP translation pathway by competing with mRNAs for RBP binding. A high level of CircPABPN1, a target circRNA of HuR, inhibits the binding of HuR to PABPN1 mRNA. Also, the extensive binding of circPABPN1 to HuR prevents HuR from binding to PABPN1 mRNA and decreases the translation of PABPN1. Overall, competition between circRNA and its homologous mRNA for RBPs affects translation [103].

## 4. Association of RNA-RBP Interactions with Human Diseases

RBPs play critical roles in multiple processes of gene expression and regulation, including RNA alternative splicing, stability, and mRNA export and translation, which are key participants in post-transcriptional events [104]. They influence the formation of mature mRNAs and subsequent protein synthesis by binding to specific sequences or structures in the RNA [105]. The interactions between RNA and RBPs create a complex network, and malfunctions in this network can be the source of many diseases. Mutations in RBPs can disrupt the homeostasis of this network, resulting in abnormalities in the RNA metabolism that contribute to various human diseases [106].

The interaction of RNA-RBPs is involved in the progression of a large number of human diseases, including neurodegenerative diseases, metabolic diseases, cardiovascular diseases, and cancerous tumors [62,107,108,109,110,111]. During the process of disease development, there is often an alteration in the binding capacity of the associated RNAs and RBPs at their binding regions. This is generally caused by post-translational modifications (PTMs) of the residues of RBPs, including acetylation, phosphorylation, ubiquitination, and N6-methyladenosine methylation [112], because the charge introduced by PTM affects the stability of the RBDs as well as the binding ability of the RNA [113]. It has been shown that RBP Sam68 acetylation is positively correlated with its ability to bind RNA, which means the binding process can be positively regulated by acetylation [114]. Enhanced Sam68 acetylation improves the binding ability of target genes that are closely related to tumor cell proliferation [115]. Proline-rich RNA-binding protein (Prrp) is a multifunctional RBP, whose lysine 150 acetylation of its RBD regulates subcellular localization and the binding activity of the two RRMs, with implications for spermatogenesis and normal cell growth [116]. Fusion sarcoma (FUS), a ubiquitously expressed RBP, whose lysine acetylation in the RRM reduces RNA binding to FUS and reduces cytoplasmic inclusion body formation, has been associated with familial amyotrophic lateral sclerosis (ALS) and frontotemporal dementia (FTD) development [117]. In addition to PTM, specific RBPs have therapeutic potential in treating diseases through other pathways, for example, the survival of motor neuron 1 (*SMN1*) gene, whose mutation or deletion is a major cause of spinal muscular atrophy (SMA) [118]. Most humans carry another gene *SMN2* that also can produce a low level of SMN protein because of the predominant skipping of exon 7 during splicing. It is manifested that changing the alternative splicing of *SMN2* can enhance the production of the functional SMN protein and potentially ameliorate the SMA phenotype [119]. These studies motivated therapeutic strategies to promote exon 7 inclusion in SMN2, which may be able to compensate for *SMN1* loss of function in SMA [120]. And Nusinersen (Spinraza), the first drug approved by the US Food and Drug Administration (FDA) for the treatment of SMA, is an antisense oligonucleotide that promotes *SMN2* exon 7 inclusion [121]. Additionally, RBPs are also involved in the pathogenesis of autoimmune diseases, where they influence the activation and function of immune cells by regulating their RNA metabolism. In multiple sclerosis (MS), aberrant localization of RBPs, such as hnRNPA1 and TDP-43, results in RNA metabolism disruption and immune cell dysfunction [122].

Owing to the abnormal function of RBPs playing a key role in the development of diseases, they can also provide new strategies for therapy. By regulating the activity or expression levels of RBPs, the expression of disease-related genes and cellular functions can be affected, leading to the treatment of diseases. For example, HuR proteins can stabilize certain mRNAs by binding to the ARE sequences of mRNAs and inhibiting the activity of HuR may help to reduce the survival of tumor cells [123]. Inhibitors targeting HuR proteins have been developed for use in studies of cancer therapy [124]. Examples of such inhibitors include MS-444, DHTS, and AZA-9, which are nanomolecular agents that inhibit HuR by targeting its RRM1 and RRM2 to block RNA-binding activity [125]. In addition, RNA mis-splicing underlies a growing number of human diseases and splicing-modulation therapy can become an emerging strategy for the treatment of related diseases [126]. As a core unit of alternative splicing, SR proteins alter their subcellular localization and function, thereby regulating pathologically alternative splicing. Splice-switching oligonucleotides (SSOs) have been approved by the FDA for Duchenne muscular dystrophy (DMD) and SMA, targeting the splice site to prevent the binding and assembly of the spliceosome to the target gene [127,128]. It has also been found that m6A modification regulates alternative splicing patterns by recruiting specific RBPs or directly affecting the interaction with the target RNA, offering a personalized approach to cancer treatment based on the integration of alternative splicing and m6A modification [129]. And therapeutic strategies targeting specific RBPs or their interactions with RNAs are being explored, such as the development of synthetic RBPs and the targeting of small molecules or oligonucleotides, which will provide new avenues for cancer treatment [130]. Accompanying further studies on the regulatory mechanisms and therapeutic potential of RBPs may reveal new therapeutic targets, which are of great relevance for the treatment of ALS, cardiac diseases, and cancers, among others [131,132].

## 5. Conclusions and Prospect

Our review points out the gene expression and regulation under the RNA-RBP action network at multiple levels, such as the interaction of different RNAs with RBPs, including the synthesis and maturation of coding RNA as well as the regulation of ncRNA. In addition, we discuss diverse ways for the regulation of gene expression, including the involvement of RNA alternative splicing, mRNA export, mRNA translation, and RNA stabilization and degradation. Moreover, there is a tight connection between disease occurrence and RNA-RBP interactions, which provides valuable insights for targeted therapy. We also illustrate the role played by the classical RBDs of RBPs in the process of binding to RNA at the molecular level based on the structural and functional characteristics of RBPs. While structural changes in the RBDs or the binding of RBPs to RNA are affected by physicochemical factors, these will disrupt the RNA-RBP action network and trigger related diseases. Therefore, a deep understanding of RNA-RBP interactions is the basis for the in-depth exploration of human disease pathogenesis and therapeutic approaches. Drugs or gene intervention strategies targeting RBPs are still in their infancy. In the future, the discovery of new RNA binding sites and relevant modification sites, as well as in-depth research on the molecular mechanisms of RBPs regulating gene expression, are important directions for enriching the codebook of therapeutic human diseases. At present, the knowledge about RBPs is enriching and deepening, giving birth to many research fields including RNA binding site detection, RBP synthesis and design, and the application mechanism of RBPs in diseases. However, we also found that there are many RBPs whose interactions with RNAs do not depend on classical RBDs, which means that their interaction mechanisms need to be further explored. In addition, among the classical RBDs, the main focus has been on the binding of the ZnF domain to DNA, with little attention paid to the ZnF domain itself. Although it is not difficult to realize that the binding of the ZnF domain to RNA is the guarantee of its function, the detailed process and mechanism need to be further studied. In conclusion, there is still a long way to go to completely clarify the complex regulatory network between RNA and RBPs, especially to explore the binding mode of RNA and RBPs and the accompanying biological processes after binding.

## Abbreviations

AGO	Argonaute
ALS	amyotrophic lateral sclerosis
AREs	AU-rich elements
CCSs	cohesin complex subunits
CPEB	cytoplasmic polyadenylation element-binding protein
DMD	duchenne muscular dystrophy
eIF	eukaryotic initiation factor
eRF	eukaryotic release factor
FDA	Food and Drug Administration
FTD	frontotemporal dementia
FUS	fusion sarcoma
hnRNP K	heterogeneous nuclear ribonucleoprotein K
HuR	human antigen R
IDRs	intrinsically disordered protein regions
IRE	iron-responsive element
IRP1	iron-regulatory protein 1
KH	hnRNP-K-Homology
KH3	the third KH domain
KH4	the fourth KH domain
KSRP	KH-type splicing regulatory protein
lncRNAs	long non-coding RNAs
miRNAs	microRNAs
MPDH	inosine 5′-monophosphate dehydrogenase
MS	multiple sclerosis
Nab2	Nuclear Poly(A) RNA-binding Protein 2
ncRNAs	non-coding RNAs
NXF1	nuclear export factor 1
PABP	Poly (A)-binding protein
PCBPs	Poly (C)-binding proteins
PRRC2B	proline-rich coiled-coil 2B
Prrp	proline-rich RNA-binding protein
PTMs	post-translational modifications
RBDs	RNA-binding domains
RBM46	RNA-binding motif protein 46
RBPs	RNA-binding proteins
RISCs	RNA-induced silencing complexes
RRMs	RNA recognition motifs
siRNAs	small interfering RNAs
SMA	spinal muscular atrophy
SMN	survival of motor neuron
SSOs	splice-switching oligonucleotides
TB-RBP	Testis–brain RNA-binding protein
ucRBPs	unconventional RBPs
ZnF	zinc finger
